# Supplementary material for: A novel true random number generator based on a stochastic diffusive memristor
Source: Nat Commun. 2017 Oct 12;8:882. doi: 10.1038/s41467-017-00869-x (PMC5638922; doi:10.1038/s41467-017-00869-x)
Supplement: Supplementary file 1 — Supplementary Information for Publishing [file 41467_2017_869_MOESM1_ESM.pdf]

Supplementary Figures

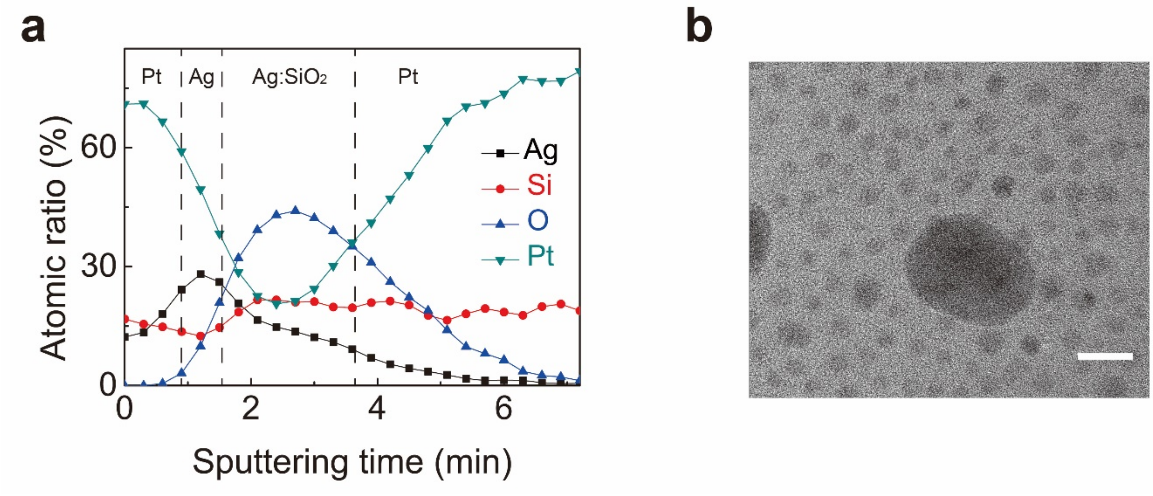

Supplementary Figure 1 | Physical characterizations of the Ag:SiO_2 film. (a) XPS depth profile of the Pt/Ag/Ag:SiO_2/Pt stack. (b) Transmission electron microscopy (TEM) bright field (BF) image of as-deposited Ag:SiO_2 thin film on a SiNx membrane. Dense Ag nanoclusters (normally 2 to 5 nm, some occasionally larger than 10 nm) are uniformly dispersed in the SiO_2 matrix. Scale bar: 10 nm.

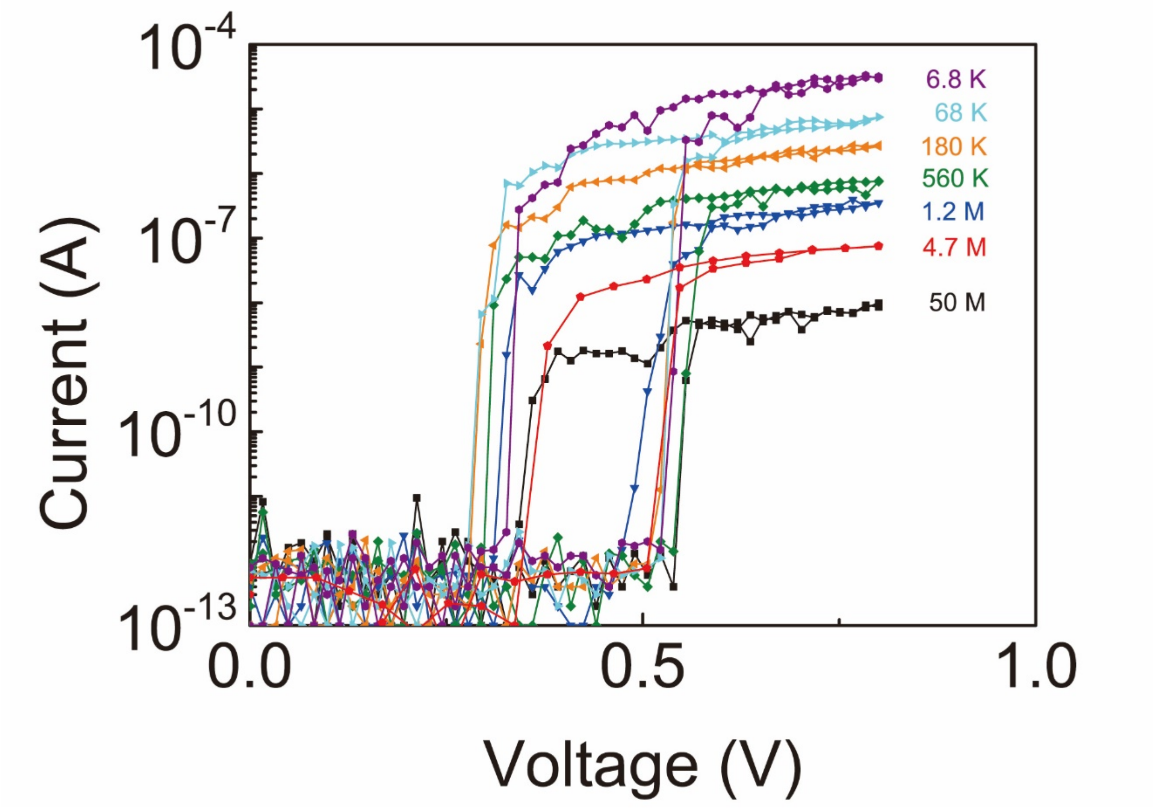

Supplementary Figure 2 | Tuning ON state current and ON/OFF window with series resistors. DC sweeps of a Ag:SiO_2 device connected in series with resistors of different resistance, indicating that a series resistor is effective in limiting the ON state current and tuning the ON/OFF window.

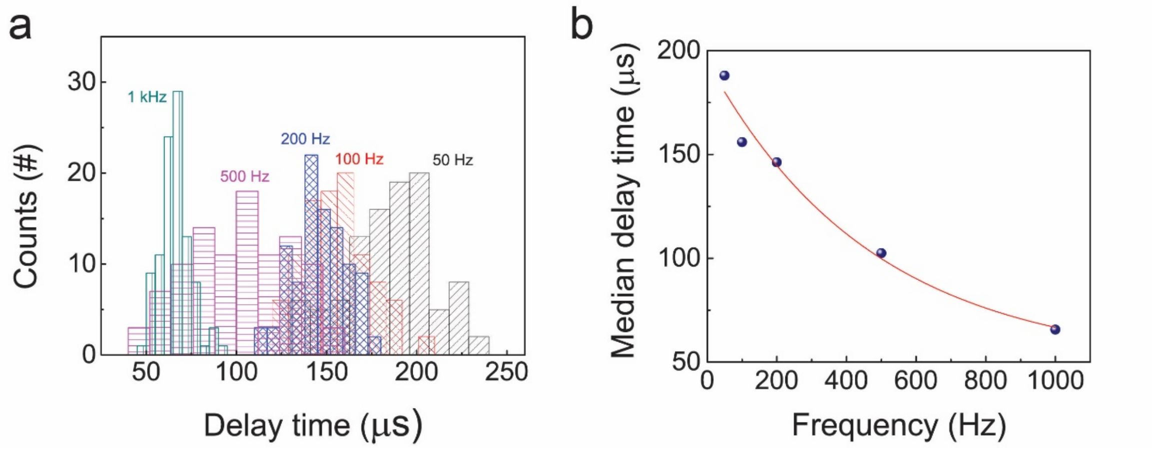

Supplementary Figure 3 | Frequency dependency of delay time. (a) Distributions of delay time at different frequencies (from 50 Hz to 1 kHz) for electrical pulses with the same voltage amplitude and pulse width (0.5 V, 300 μs). (b) Plot of median delay time vs. frequency. A higher frequency leads to a shorter median delay time.

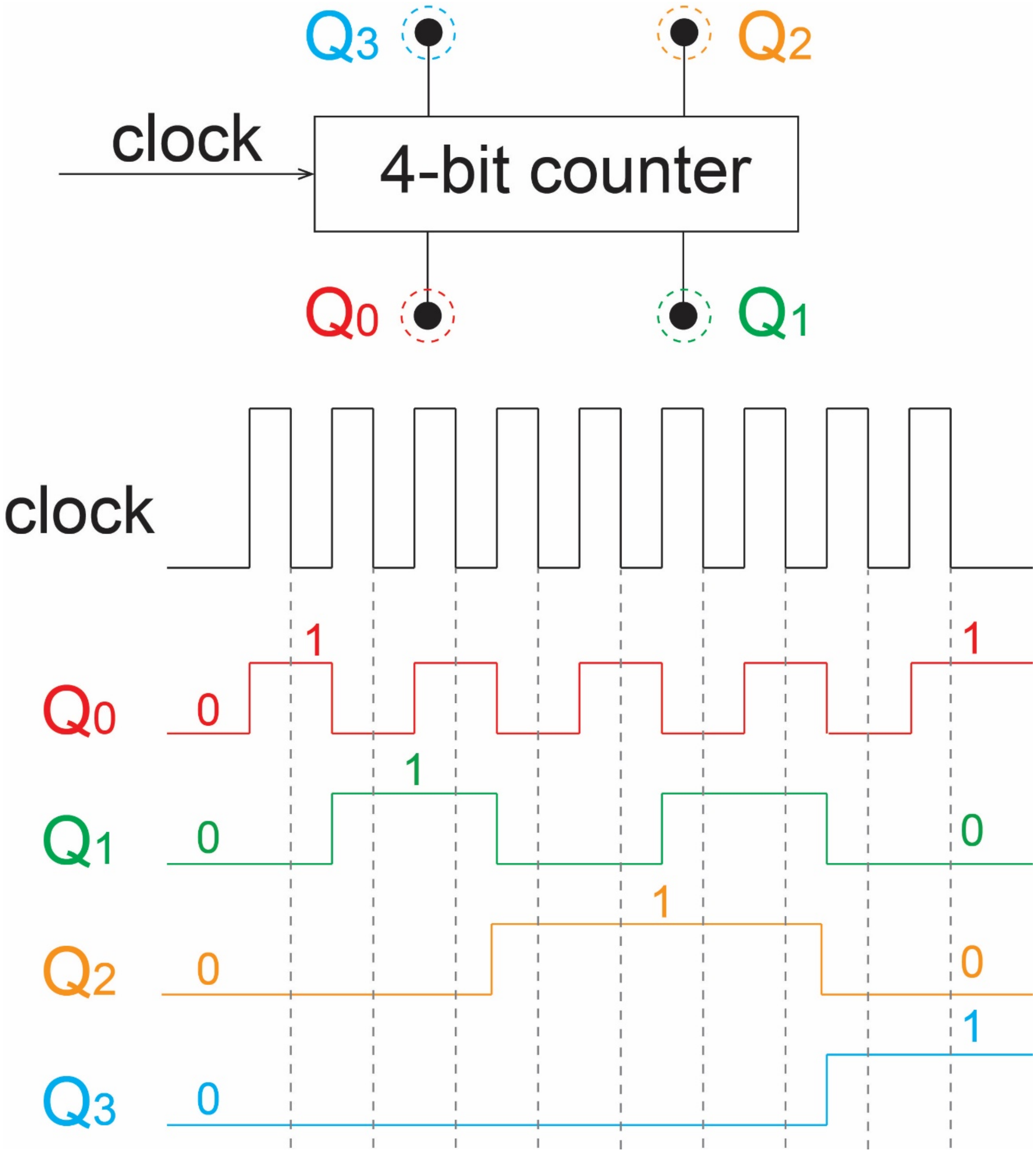

Supplementary Figure 4 | Working principle of a multi-bit counter. Here we use a 4-bit counter as an example. The counter can each time output a 4-bit binary value (Q_3 Q_2 Q_1 Q_0), where Q_0 has the lowest order. In response to the input clock signal, different-order bits flip in different frequencies and the lowest-order bit (Q_0) flips in the highest frequency. Starting from (0 0 0 0), after receiving 9 clock pulses, the counter will output (1 0 0 1). The details of how those bit flips are schematically shown here.

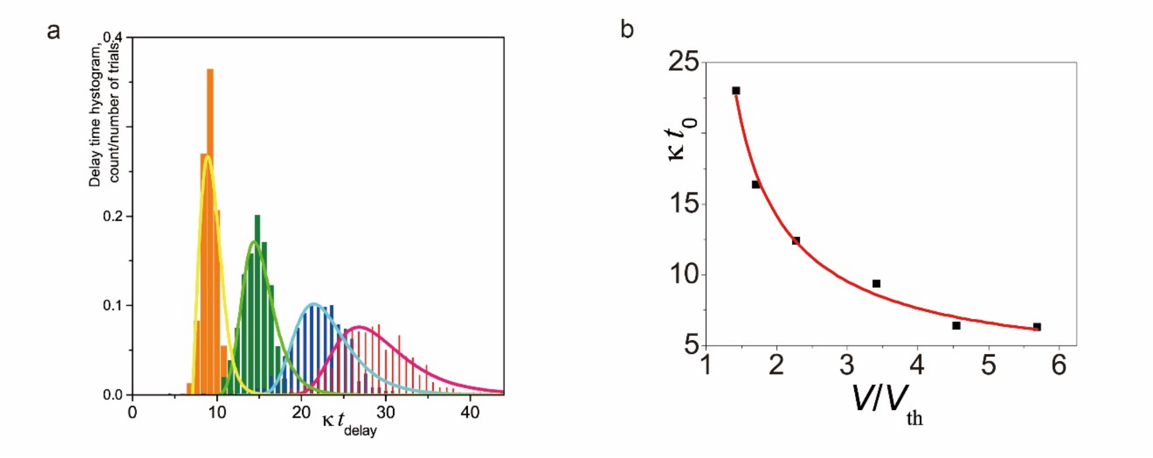

Supplementary Figure 5 | Statistics of delay time from nanoparticle dynamical simulations. (a) Simulated distributions (normalized by the total number of pulses) of delay time under different voltages and (b) corresponding fitting curve of t_0 according to eq. 3 in main text (the fitting parameter is $V_{th,}$ while κt_1= 5, $\kappa\tau$= 16 was fixed to be the same as in all simulations). Here we used the following voltage pulse parameters (the same as in fig 4a-e): voltage pulse duration κt_p= 80 (allowing enough time to switch to low resistive state for every pulse), inter-pulse interval $\kappa\Delta t$= 360 (allowing enough times to relax) and voltage amplitude V_{am}/V_{th}= 1.6 (same as fig. 4 a-e), 2.2, 3.3, 5.5; potential versus temperature as in fig. 4f. The delay time becomes shorter with the increase of applied voltage amplitudes and the distribution becomes narrower and the time shift becomes smaller, consistent with experiment data shown in Fig. 1d.

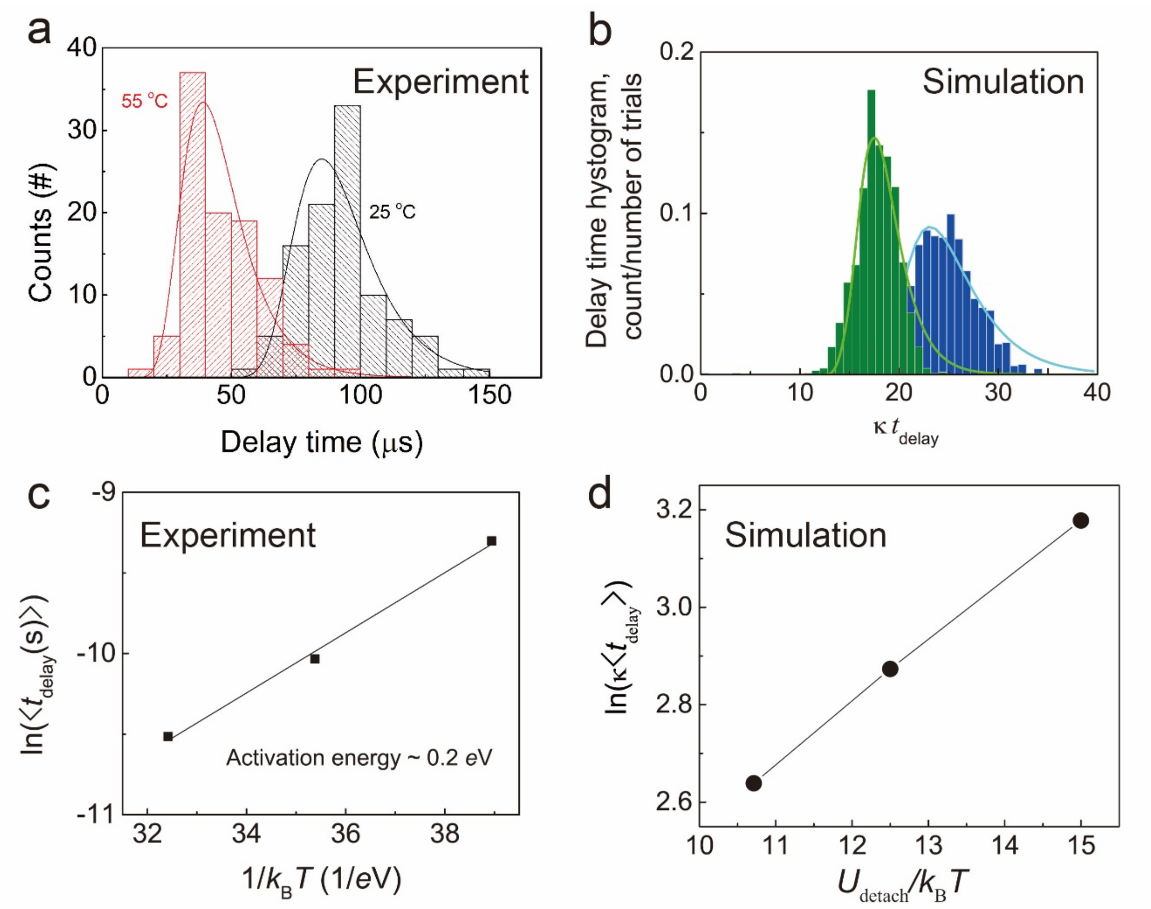

Supplementary Figure 6 | Temperature dependence of delay time from experiments and simulations. (a) Distributions of delay time (t_{delay}) at 25 ºC and 55 ºC (voltage amplitude: 0.5 V and pulse width: 300 μs @ 1 kHz) can still be fitted by equation (2) in the main text, in perfect agreement with (b) simulated results (all parameters are the same as in simulations presented in the main text and temperature ratio for blue and green histograms are 5/6). (c) The delay time is decreasing as the temperature increases with an activation energy of 0.2 *e*V, also consistent with that in simulations in (d).

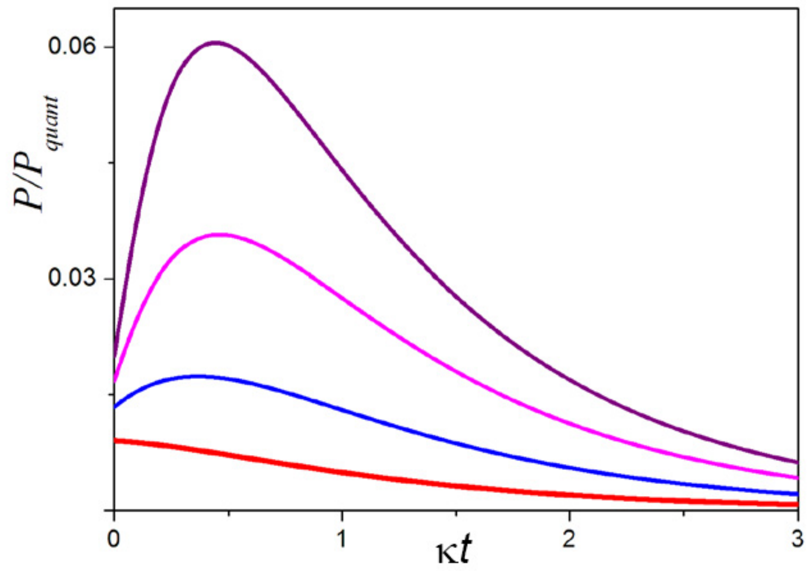

Supplementary Figure 7 | The distribution of the delay time in quantum regime. Red: $\frac{\hbar\omega}{2k_{\mathrm{B}}T}$=0.6; blue: $\frac{\hbar\omega}{2k_{\mathrm{B}}T}$=1; magenta: $\frac{\hbar\omega}{2k_{\mathrm{B}}T}$=1.5; brown: $\frac{\hbar\omega}{2k_{\mathrm{B}}T}$=2. Note that quantum fluctuations result in the maximum of the distributions shifting to the right (we used $U_0/(\hbar\omega/2) = 6$). The probability density is normalized by $\sqrt{\frac{\pi\hbar}{m\omega}}$.

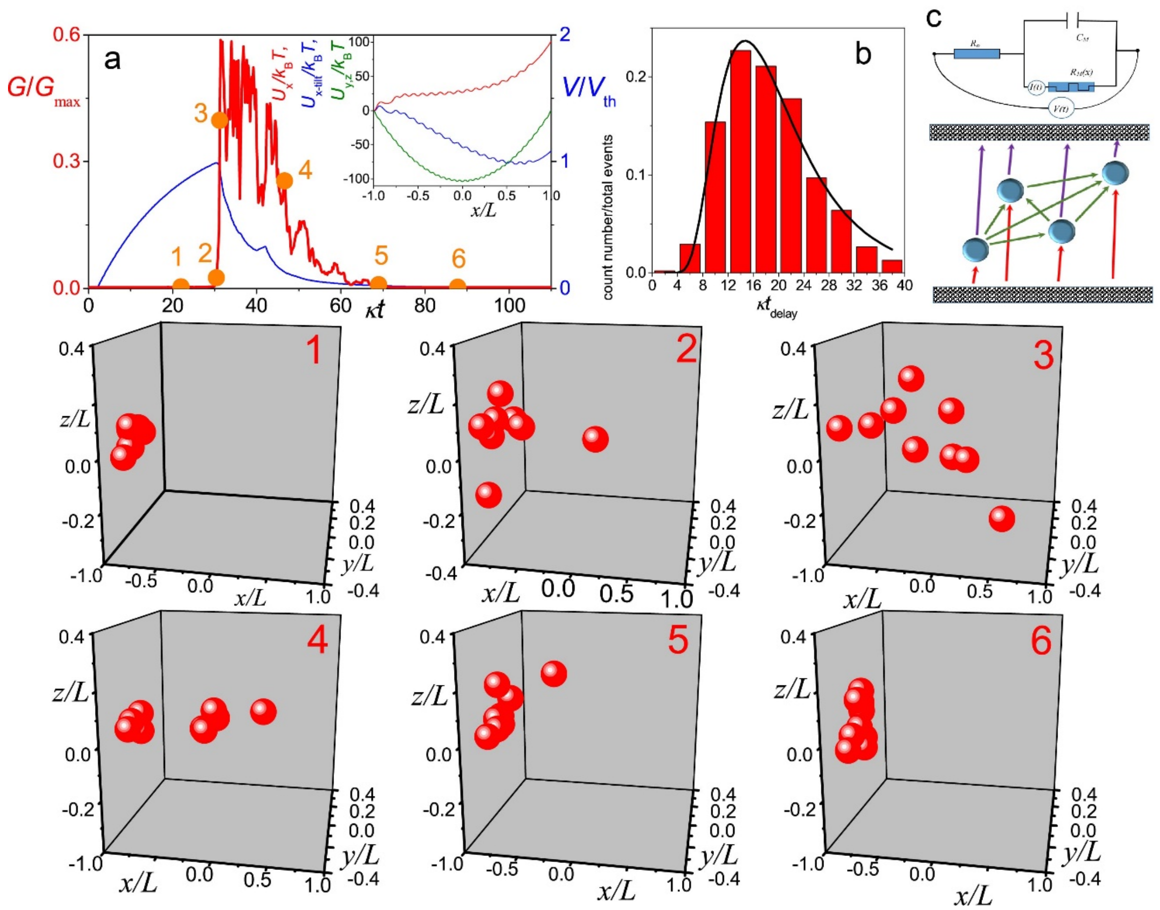

Supplementary Figure 8 | 3D simulations of a diffusive memristor dynamics. (a) Conductance (red curve) normalized by its maximum value and voltage across the memristor normalized by its threshold V_{th}, potentials used in simulations are shown in the inset (see Supplementary Note 3). Simulations are done for 9 Ag-nanoparticles. (b) The histogram is fitted by the analytically derived distribution, the fitting is quite good despite the complex 3D potential. Simulations are done for 6 Ag nanoparticles. (c) The possible current paths between memristor electrodes via Ag nanoparticles (for simplicity the case of only 4 Ag nanoparticles are shown). Panels 1-6 show the Ag particles positions at the points marked by orange circles in panel a), panels 1-3 demonstrate how the 3D conducting path are forms, while panels 4-6 show the relaxation of the device to its off state. The ratio λ/L=0.2, all other parameters are the same as in 1D simulations.

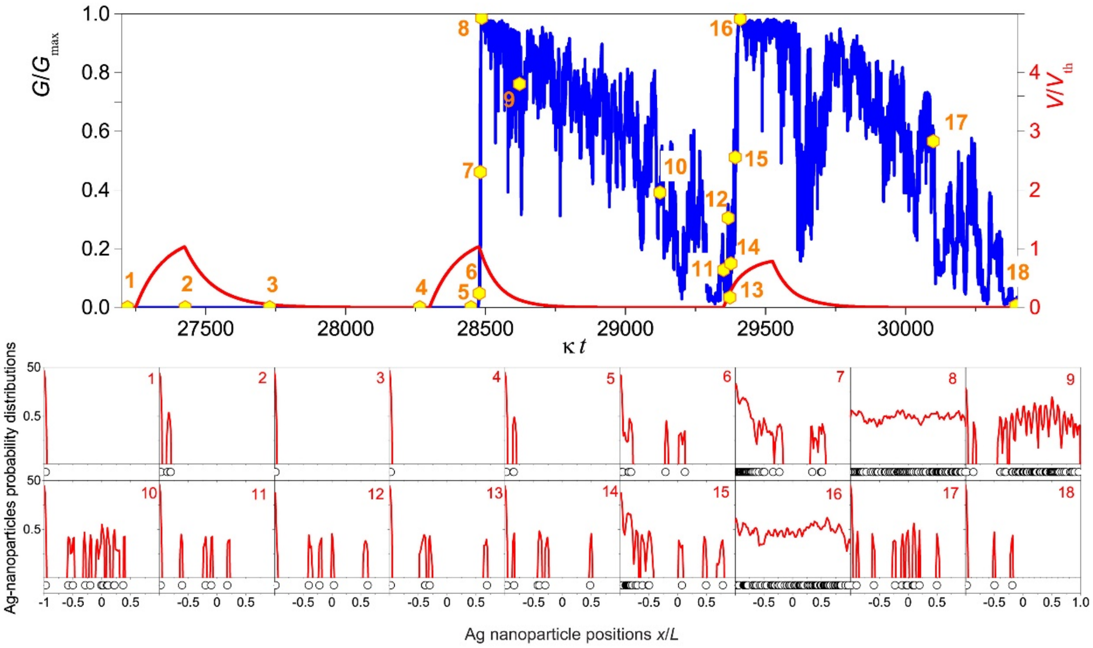

Supplementary Figure 9 | Simulated three possible cases during continuous pulse switching: no switching, successful random number generation, and failure due to incomplete relaxation. The results show device conductance change (in blue) in response to three pulse cycles (in red) and corresponding evolution of Ag nanoparticle distribution. Here we used the following voltage pulse parameters: voltage pulse duration κt_p=35, inter-pulse interval $\kappa\Delta t$=140 and voltage amplitude V_{am}/V_{th}=1.6; potential versus temperature as in fig. 4f. During the first pulse, the system had not enough time to be excited to the low resistive state (see main panel and subpanels 1-4), thus the random number was not generated. Note that several Ag particles are detached from the Ag-electrode and form a large cluster nearby (panel 2), it was not enough time to particles escape the cluster and unfold; and the particles are absorbed by the electrode as soon as pulse are over (panel 3). By the second shown input pulse arrival, the device is well relaxed (panel 4), when voltage gradually increases, Ag-particles first detached from the Ag-electrode and form the big cluster (panel 5), then cluster elongates and several Ag-nanoprticles starts travelling (panel 6) towards the other electrode starting positive feedback (lowering resistance results in generation more heating, higher temperature detached more particles from the Ag electrodes and push away from the big cluster, thus lowering resistance even further, panels 7). Finally, the memristors turned ON (panel 8) after some delay time. In this case, the random number is successfully generated. After the pulse is off, the device starts relaxing back to its high resistive state (first by breaking continuous conducting path panel 9, then, fragmenting into many small Ag-clusters, panel 10, which tends to be absorbed by the Ag electrode). However, since some Ag-nanoparticles are not absorbed and are still travelling between electrodes (panel 11) when the next pulse arrives and, thus, the device doesn't have enough time to settle in its high resistive state. In this case, the memristor is switched to ON state gradually with no delay time (Panels 12-18) during the voltage increase.

Moreover, the conductance can increase non-monotonically when voltage is low due to fluctuations in Ag-cluster numbers and distance between them (panels 12-14); the conductance starts to relatively fast increase when voltage is high enough to allow Ag-particles to escape (panel 15) from the large cluster near Ag-electrodes, forming in the end conducting path (panel 16). In this case, failure to generate random bits occurs.

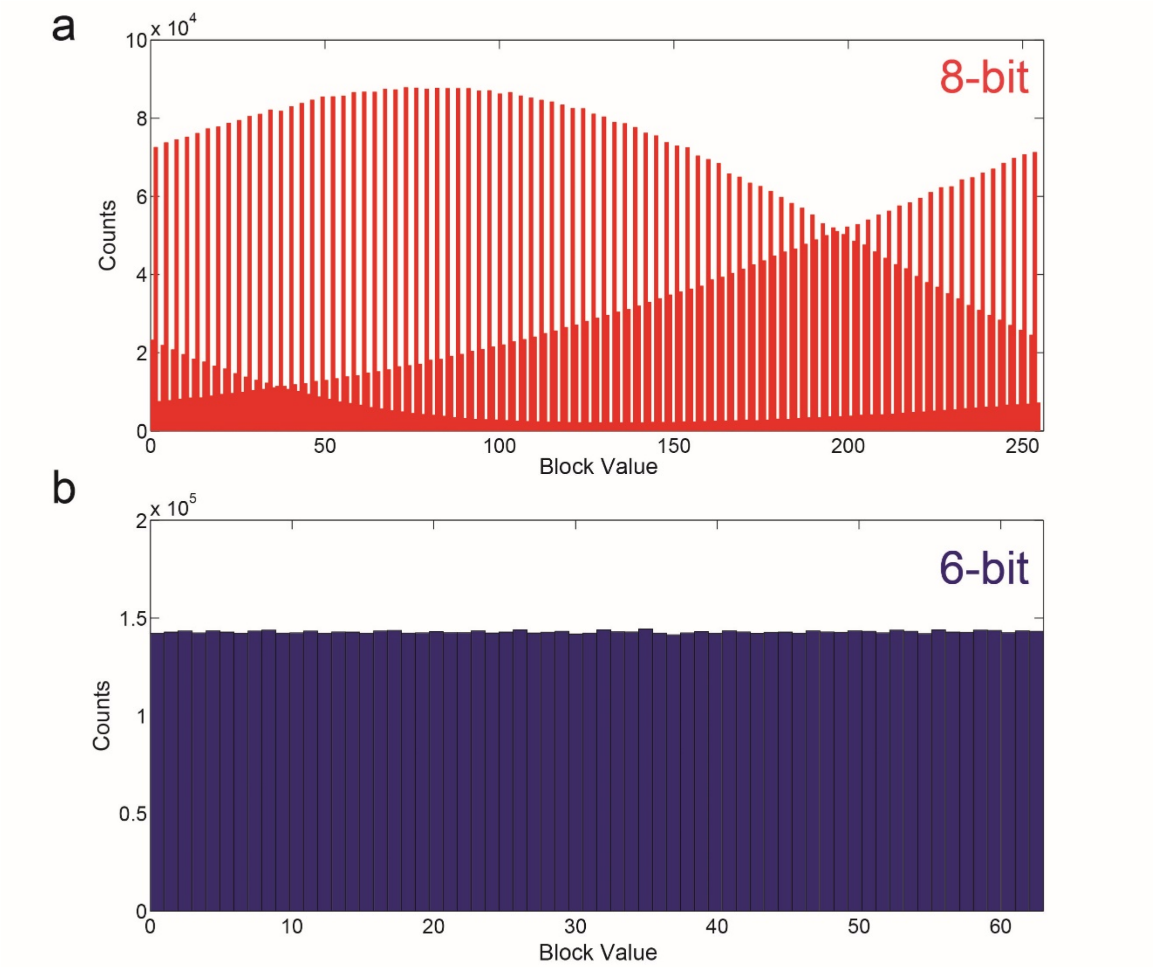

Supplementary Figure 10 | Bit frequency uniformity at different data collection scheme. (a) If each time 8-bit data is collected from the 8 lower-order bits of the counter, the block counts are not uniform. (b) If only the 6 lower-order bits are collected, each block is equally likely. The data collecting circuits uses a built-in clock in the micro-controller that has a 11.0592 MHz crystal oscillation frequency. The 8th lowest bit flips 4 times slower than the 6th lowest bit and hence the experiment suggests that ~ 4 times faster clock signal (> 44.2368 MHz) is required for an 8-bit counter.

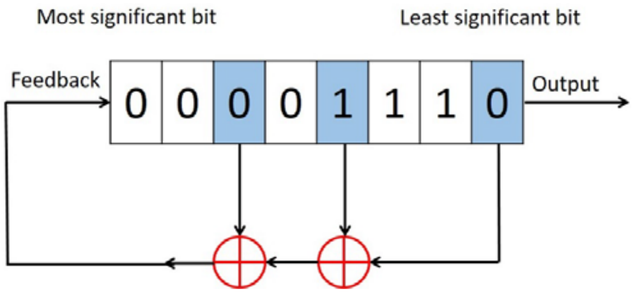

Cycle	Current state in the shift registers								Output	Feedback
**1**	0	0	0	0	1	1	1	0	0	1
**2**	1	0	0	0	0	1	1	1	1	1
**3**	1	1	0	0	0	0	1	1	1	1
**4**	1	1	1	0	0	0	0	1	1	0
**5**	0	1	1	1	0	0	0	0	0	1
**Re-seed techniques apply a new seed 10110100**										
**17**	1	0	1	1	0	1	0	0	0	0
**18**	0	1	0	1	1	0	1	0	0	1
**19**	1	0	1	0	1	1	0	1	1	1
**20**	1	1	0	1	0	1	1	0	0	0

Supplementary Figure 11 | The working principal of a combination of TRNG and a linear-feedback shift register (LFSR) to increase the bitrate. The initial value of the LFSR is called the seed, and the bits to which the XOR gates are connected are called the tap bits. Here the taps are at positions [1, 4, 6]. At the first step, an 8-bit sequence (00001110) is generated from our TRNG and is used to seed the LFSR. The LFSR will start working, triggered by the clock edges. All the bits in the current state will be shifted to the right and the least significant bit (in this case, “0”) will be the output. At the same time, tap bits (“0”, “1” and “0”) will be XORed together and resulting “1” is used for feedback and input to the most significant bit. The table lists how the bits flip for the first 5 cycles of this LFSR, which will continue generating bits indefinitely. However, to ensure unpredictability, we need to re-seed the LFSR before the pattern repeats. In this example, we refresh the LFSR with a new 8-bit sequence (10110100) generated from the diffusive memristor TRNG after 16 cycles. The LFSR will then continue running based on the new initial state as shown in the table.

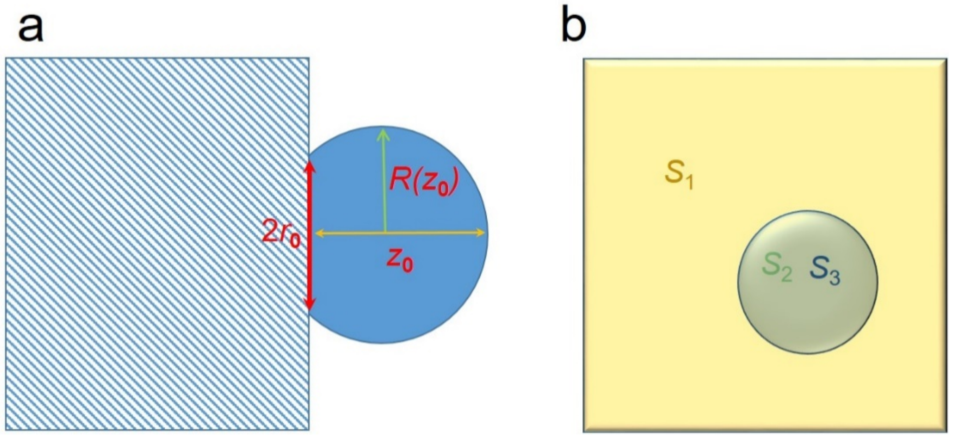

Supplementary Figure 12 | Sketch of an Ag nanoparticle detaching from the Ag electrode. (a) The shape of the particle varies during the detaching process in order to minimize its surface energy. We describe the shape of particles in cylindrical coordinates ($\rho(z)$, z), $\rho(z=0) = \rho_0$ (no dependence of ρ on the angular coordinate was assumed). The total "height" z_0 of the particle and its effective instantaneous radius R are shown on the sketch. (b) The free electrode surface S_1 increases when particle detaching; the contact surface S_2 between nanoparticle and the electrode decreases as particle departing, while the free surface of the particle itself S_3 increases in this process. In the estimate below, it was assumed that the interfacial energy γ per unit area for interfaces S_1 and S_3 being the same, while the interfacial energy γ_1 per unit area for the interface S_2 can vary from 0 (clean interface) to 2γ (several SiO_2 layer separating two contacting Ag surfaces).

Supplementary Tables

	P -Value	Pass Rate	min. pass rate	SUCCESS/ FAILURE
1.Approximate Entropy	0.051942	49/50	47/50	SUCCESS
2.Block Frequency	0.171867	50/50	47/50	SUCCESS
3.Cumulative Sums	0.739918, 0.153763	96/100	94/100	SUCCESS
4.FFT	0.051942	49/50	47/50	SUCCESS
5.Frequency	0.935716	48/50	47/50	SUCCESS
6.Linear Complexity	0.955835	49/50	47/50	SUCCESS
7.Longest Run	0.779188	50/50	47/50	SUCCESS
8.Non Overlapping Template	-	7358/7400	6956/7400	SUCCESS
9.Overlapping Template	0.657933	50/50	47/50	SUCCESS
10.Random Excursions	-	195/208	192/208	SUCCESS
11.Random Excursions Variant	-	460/468	432/468	SUCCESS
12.Rank	0.494392	49/50	47/50	SUCCESS
13.Runs	0.191687	49/50	47/50	SUCCESS
14.Serial	0.262249, 0.026948	98/100	94/100	SUCCESS
15.Universal	0.494392	50/50	47/50	SUCCESS

Supplementary Table 1 | Randomness test (NIST 800-22 test suite) results for the produced 50 M binary bits from a combination of 16-bit LFSR and our diffusive memristor TRNG, suggesting the bitrate can be easily increased from 6 kbs^{-1} to 300 kbs^{-1} without affecting the performance.

	P -Value	Pass Rate	min. pass rate	SUCCESS/ FAILURE
1.Approximate Entropy	0.275709	11/11	9/11	SUCCESS
2.Block Frequency	0.090936	11/11	9/11	SUCCESS
3.Cumulative Sums	0.834308, 0.637119	11/11, 11/11	9/11	SUCCESS
4.FFT	0.025193	11/11	9/11	SUCCESS
5.Frequency	0.162606	11/11	9/11	SUCCESS
6.Linear Complexity	0.437274	10/11	9/11	SUCCESS
7.Longest Run	0.437274	11/11	9/11	SUCCESS
8.Non Overlapping Template	-	1608/1628	1332/1628	SUCCESS
9.Overlapping Template	0.834308	11/11	9/11	SUCCESS
10.Random Excursions	-	72/72	64/72	SUCCESS
11.Random Excursions Variant	-	156/162	144/162	SUCCESS
12.Rank	0.637119	11/11	9/11	SUCCESS
13.Runs	0.090936	10/11	9/11	SUCCESS
14.Serial	0.637119, 0.437274	11/11, 11/11	9/11	SUCCESS
15.Universal	0.637119	10/11	9/11	SUCCESS

Supplementary Table 2 | Randomness test (NIST 800-22 test suite) results for a diffusive memristor true random number generator working at 85 ^{o}C. Total 11 M binary bits were collected and passed all the 15 tests with no post processing. Compared to operation at room temperature, the bitrate decreased to 3 kbs^{-1} since we can only collect the 3 lower-order bits from the counter with the same 11.0592 MHz crystal oscillation frequency. The bitrate can be kept at 6 kbs^{-1} if ~ 88.4736 MHz clock signal is used.

	P -Value	Pass Rate	min. pass rate	SUCCESS/ FAILURE
1.Approximate Entropy	0.383827	54/54	51/54	SUCCESS
2.Block Frequency	0.534146	54/54	51/54	SUCCESS
3.Cumulative Sums	0.699313, 0.616305	53/54, 53/54	51/54	SUCCESS
4.FFT	0.657933	54/54	51/54	SUCCESS
5.Frequency	0.657933	53/54	51/54	SUCCESS
6.Linear Complexity	0.171867	53/54	51/54	SUCCESS
7.Longest Run	0.534146	54/54	51/54	SUCCESS
8.Non Overlapping Template	-	7921/7992	7548/7992	SUCCESS
9.Overlapping Template	0.236810	54/54	51/54	SUCCESS
10.Random Excursions	-	296/296	272/296	SUCCESS
11.Random Excursions Variant	-	661/666	612/666	SUCCESS
12.Rank	0.494392	11/11	51/54	SUCCESS
13.Runs	0.090936	54/54	51/54	SUCCESS
14.Serial	0.574903, 0.108791	53/54, 53/54	51/54	SUCCESS
15.Universal	0.213309	54/54	51/54	SUCCESS

Supplementary Table 3 | Randomness test (NIST 800-22 test suite) results for 54 M binary bits from a single diffusive memristor with a bitrate of 6 kbs^{-1} at room temperature.

First 2 M Bits			Last 2 M Bits		
	P -Value	SUCCESS/ FAILURE	Last 2 M	P -Value	SUCCESS/ FAILURE
1.Approximate Entropy	0.285	SUCCESS	1.Approximate Entropy	0.924	SUCCESS
2.Block Frequency	0.94	SUCCESS	2.Block Frequency	0.788	SUCCESS
3.Cumulative Sums	0.388, 0.187	SUCCESS	3.Cumulative Sums	0.275, 0.235	SUCCESS
4.FFT	0.881	SUCCESS	4.FFT	0.058	SUCCESS
5.Frequency	0.201	SUCCESS	5.Frequency	0.935	SUCCESS
6.Linear Complexity	0.314	SUCCESS	6.Linear Complexity	0.195	SUCCESS
7.Longest Run	0.297	SUCCESS	7.Longest Run	0.078	SUCCESS
8.Non Overlapping Template	-	SUCCESS	8.Non Overlapping Template	-	SUCCESS
9.Overlapping Template	0.638	SUCCESS	9.Overlapping Template	0.106	SUCCESS
10.Random Excursions	-	SUCCESS	10.Random Excursions	-	SUCCESS
11.Random Excursions Variant	-	SUCCESS	11.Random Excursions Variant	-	SUCCESS
12.Rank	0.367	SUCCESS	12.Rank	0.309	SUCCESS
13.Runs	0.413	SUCCESS	13.Runs	0.585	SUCCESS
14.Serial	0.202, 0.081	SUCCESS	14.Serial	0.692, 0.763	SUCCESS
15.Universal	0.452	SUCCESS	15.Universal	0.352	SUCCESS

Supplementary Table 4 | Randomness test (NIST 800-22 test suite) results for first 2 M first 2 M bits (of the 54 M bits from the same diffusive memristor) from the very beginning cycles and the last 2 M bits from the same device that after ~ 9×10^6 cycles. Both of them passed the tests, indicating that even after many cycles the randomness in memristive switching remains sufficient to produce high quality random bits using our method.

Supplementary Notes

Supplementary Note 1 | Random escape time from deep potential well of interfacial energy (detaching time of Ag nanoparticle from reservoir)

Interfacial barrier

First we consider how an Ag-nanoparticle detaches from the boundary. We assume that its mass/volume does not changes during the detachment (no material exchange between the electrode and the particle), but its shape is adjusting to minimize the interfacial energy. We will use cylindrical coordinates (assuming no dependence of particle shape on azimuthal angle), the Ag-electrode plane is at z=0, and the Ag nano-particle is attached to the electrode by surface $S_0 = \pi\rho_0^2$, thus, $\rho(z=0) = \rho_0$ (Supplement Fig. 12a). There are three different interfaces (Supplement Fig. 12b) which areas are changing when the Ag-nanoparticle detaching from the electrode: the free surface S_1 of the Ag-electrode (not contacting to the nanoparticle), the contact surface S_2 between nanoparticle and the electrode, and the surface S_3 of the nanoparticle itself (not contacting to the electrode). All interfaces can have different interfacial energies per unit area, but, for our simple estimates, we assume that the surface energy γ per unit area of interfaces S_1 and S_3 is the same. The interfacial energy γ_1 of the interface S_2 can vary from zero (if no any other materials is between the Ag-nanoparticle and the electrode – very clean interface) to 2γ if the two contacting surfaces are separated by several layers of SiO_2.

The interfacial energy can be estimated as $U_{\text{det}} = \gamma S_3 - \gamma\pi\rho_0^2 + \gamma_1\pi\rho_0^2$ where the surface S_3 of the interface S_3 changes with particle shape as follows $S_3 = \int_0^{z_0} 2\pi\rho\sqrt{1+(\rho')^2}\,dz$. The second term in the equation for U_{det} originates from the

surface energy $\gamma(A - \pi\rho_0^2)$ of the Ag-electrode (where A is the whole electrode area and, the constant energy γA in the expression for potential energy U_{det} can be omitted). The last term in U_{det} is the interfacial energy of the contact surface of the Ag-nanoparticle and the Ag-electrode. In order to calculate U_{det}, we have to obtain ρ, which corresponds to the minimum surface at a fixed volume of the detaching particle, thus, we need to minimize functional $F = \int_0^{z_0} 2\pi\rho\sqrt{1 + (\rho')^2}\, dz - \Lambda(\int_0^{z_0} \pi\rho^2\, dz - V_{\mathrm{Ag}})$, where Λ is a Lagrangian multiplier and V_{Ag} is the volume of the Ag-nanoparticle. Similar approach has been used to estimate surface energy earlier[1], but we modified it for the purpose of this article. Minimization conditions result in $\partial F/\partial z_0 = 0, \partial F/\partial\Lambda = 0$ and $\delta F/\delta\rho = 0$, which can be rewritten as

$$\sqrt{1 + (\rho')^2} = \frac{R}{\rho}, \qquad \rho(z_0) = 0, \qquad \int_0^{z_0} \pi\rho^2\, dz = V_{Ag}\ ,$$

where $R = 2/\Lambda$ has a simple geometrical interpretation as an instantaneous cluster radius and $\rho(0) = \rho_0$ is the radius of electrode-nanoparticle contacting area. Solution of differential equation for ρ can be written as $\rho^2 = \rho_0^2 - z^2 \pm 2\sqrt{R^2 - \rho_0^2}$, with $z_0 = R \pm \sqrt{R^2 - \rho_0^2}$. Simple but cumbersome calculations result in parametric dependence of the detaching energy U_{det}on the center mass position $x_0 = \frac{1}{V_{Ag}}\int_0^{z_0} \pi z\rho^2 dz$ of the Ag-nanoparticle: $U_{\mathrm{det}}(x_0) = \pi\gamma z_0^2 + \frac{\pi\gamma_1}{3}\left(\frac{(2r_{\mathrm{Ag}})^3}{z_0} - z_0^2\right)$, $x_0 = \frac{z_0^4}{48r_{\mathrm{Ag}}^3} + \frac{1}{3}z_0$, where $r_{\mathrm{Ag}} = \sqrt[3]{3V_{\mathrm{Ag}}/4\pi}$. The potential barrier, which the Ag-nanoparticle should overcome to detach from the electrode, can be estimated as: $\Delta U = U_{\mathrm{det}}(x_0 = r_{\mathrm{Ag}}) - \min_{x_0}[U_{\mathrm{det}}(x_0)] =$

$4\pi\gamma r_{\mathrm{Ag}}^2\left(1-\sqrt[3]{\frac{3}{4}\left(\frac{\gamma_1}{\gamma}\right)^2-\frac{1}{4}\left(\frac{\gamma_1}{\gamma}\right)^3}\right)$. The interfacial energy for Ag-SiO_2 interface can be estimated as 0.82 N/m (Ref. 2). However, in order to get an estimate for ΔU, some physical assumption should be done about detaching cluster radius and quality of interface between two contacting Ag surface (that is ratio γ_1/γ). It looks unrealistic that the large Ag-nanoparticle of radius $r_{\mathrm{Ag}} = 2\ nm$ can detached as a whole from the clean interface since the detaching barrier is too high (~ 0.26 keV). If we assume that a large nanoparticle of radius $r_{\mathrm{Ag}} = 2\ nm$ detached as a whole from a dirty S_2 interface (e.g., $\frac{\gamma_1}{\gamma} = 1.96$), we have $\Delta U \sim 0.1\ eV \sim 4 k_{\mathrm{B}} T_{\mathrm{room}}$ (here $T_{\mathrm{room}} = 300$ K). It should be noted that to fully understand the barrier would require detailed experiments that are beyond the scope of this paper.

Delay time distribution

To estimate the distribution of time needed for an Ag-nanoparticle to escape from a deep interfacial potential well in a wide temperature range (from low temperatures where quantum fluctuations play an important role), we consider a solution of the Quantum Fokker-Planck equation[3]:

$$\frac{\partial \mathcal{P}}{\partial t} = \frac{\partial}{\partial x}\left(\frac{\partial U_{\mathrm{q}}}{\partial x}\mathcal{P} + k_{\mathrm{B}} T_{\mathrm{q}} \frac{\partial \mathcal{P}}{\partial x}\right) \qquad \text{(Supplementary Equation 1)}$$

for an Ag-nanoparticle sitting at t=0 in the minima of parabolic potential $U(x) = kx^2/2$ with curvature k. Equation (Supplementary Equation 1) allows to accommodate both thermal noise and quantum fluctuations if we introduce the effective potential $U_{\mathrm{q}} = k(x^2 + \langle \delta x^2 \rangle)/2$ and temperature $k_{\mathrm{B}} T_{\mathrm{q}} = \frac{\hbar\omega}{2} \coth \frac{\hbar\omega}{2 k_{\mathrm{B}} T}$ with $\langle \delta x^2 \rangle = \hbar/2m\omega$ and $\omega =$

$\sqrt{k/m}$ (here m is mass of Ag nano-clusters). To accommodate quantum uncertainty, we assume that the particle was in its ground state at t=0: $\mathcal{P}(x, t = 0) = \left(\frac{m\omega}{\hbar\pi}\right)^{1/2} e^{-m\omega x^2/\hbar}$ (note that the solution will be very similar if we use any other Gaussian distribution as the initial state). Here, $\mathcal{P}$ is the probability density describing probability to find a particle at time t in the interval [x, x+dx]. The solution of (Supplementary Equation 1) corresponding to this problem can be written as: $\mathcal{P}(x, t) = \left(\sqrt{m\omega \tanh\frac{\hbar\omega}{2k_\mathrm{B}T} / \hbar\pi\sigma(t)}\right) \exp(-kx^2/\hbar\omega \coth\frac{\hbar\omega}{2k_\mathrm{B}T} \sigma(t))$ with $\sigma(t) = 1 - e^{-2k(t-t_\mathrm{q})}$ with $t_\mathrm{q} = \ln\left(1 - \tanh\frac{\hbar\omega}{2k_\mathrm{B}T}\right)/2k$. The probability to escape to the left from the finite (truncated) parabolic well of depth U_0 can be estimated as $\mathbb{P}(t) = \int_{\tilde{x}}^{\infty} \mathcal{P}(x, t)dx$ with $\tilde{x} = \sqrt{2U_0/k}$. Instead of calculations this integral, we can integrate the Fokker-Planck equation over the spatial coordinate from $\tilde{x}$ to infinity, resulting in

$$\frac{\partial\mathbb{P}}{\partial t} = -\left(k\tilde{x}\mathcal{P}(\tilde{x}, t) + k_\mathrm{B}T_\mathrm{q}\frac{\partial\mathcal{P}(\tilde{x},t)}{\partial\tilde{x}}\right) = \frac{k\tilde{x}}{e^{2k(t-t_\mathrm{q})}-1}\mathcal{P}(\tilde{x}, t) \quad \text{(Supplementary Equation 2)}$$

The probability of the particle to escape between time moments t, and t+dt can be written as $\mathrm{dt}\partial\mathbb{P}/\partial t$. On the other hand, the same probability is determined by the escape time distribution, that is $P_\mathrm{q} = \partial\mathbb{P}/\partial t$. Finally, using the expression for $\mathcal{P}$, we derive equation (Supplementary Equation 3) that

$$P_\mathrm{q}(\mathrm{t}) = \frac{C_\mathrm{q} e^{-\frac{A_\mathrm{q}}{1-e^{-2k(t-t_q)}}}}{\sqrt{1-e^{-2k(t-t_\mathrm{q})}}}\left(e^{2k(t-t_\mathrm{q})} - 1\right)^{-1} \quad \text{(Supplementary Equation 3)}$$

where we introduce the following parameters: $A_q = U_0/(\hbar\omega \coth\frac{\hbar\omega}{2k_\mathrm{B}T})$, $C_\mathrm{q} = \sqrt{\frac{m\omega}{\pi\hbar}\tanh\frac{\hbar\omega}{2k_\mathrm{B}T}}$. Note that at classical limit ($\hbar\omega \ll k_\mathrm{B}T$) the equation reduces to probability ($P(t) = \frac{Ce^{-\frac{A}{1-e^{-2kt}}}}{\sqrt{1-e^{-2kt}}}(e^{2kt}-1)^{-1}$) used in main text. Supplementary Fig. 7 illustrates what time delay distributions one can expect at low temperatures when quantum fluctuations start to play an essential role. Since we observed a shift of the probability distribution maximum to the left (in Supplementary Fig. 6), which contradicts to the simulated results in Supplementary Fig. 7, we believe that quantum effects are not dominant for the described device at temperatures around 300 K, but can be important at lower temperatures.

Supplementary Note 2 | RC effect introduces deterministic shift to the delay time

Due to the RC effect in real experiments, the voltage across the device is approaching to its stationary value exponentially $V(t) = V(1 - e^{-t/\tau_0})$ with characteristic "RC-time" τ_0 and the pulse amplitude V. On the other hand, the memristor can switch to its low resistance state if $V(t) > V_\mathrm{tr}$, thus, resulting in a deterministic delay

$$t_\mathrm{RC} = -\tau_0 \ln(1 - V_\mathrm{tr}/V) \quad \text{(Supplementary Equation 4)}$$

which diverges to infinity for $V \to V_\mathrm{tr}$ and decrease as $t_\mathrm{RC} = \tau_0 V_\mathrm{tr}/V$ for $V \gg V_\mathrm{tr}$.

Supplementary Note 3 | 3D nanoparticle simulations of diffusive memristors

To analyze to what extend diffusion in higher dimensions is important in the analysis of our experimental data we perform 3D simulations. We used the following Langevin equations for nanoparticles particles:

$$\eta \frac{dx_i}{dt} = -\frac{\partial U(x_i,y_i,z_i)}{\partial x_i} + \alpha \frac{V(t)}{L} + \sqrt{2\eta k_{\mathrm{B}} T}\zeta_{i,x} \qquad \text{(Supplementary Equation 5)}$$

$$\eta \frac{dy_i}{dt} = -\frac{\partial U(x_i,y_i,z_i)}{\partial y_i} + \sqrt{2\eta k_{\mathrm{B}} T}\zeta_{i,z} \qquad \text{(Supplementary Equation 6)}$$

$$\eta \frac{dz_i}{dt} = -\frac{\partial U(x_i,y_i,z_i)}{\partial z_i} + \sqrt{2\eta k_{\mathrm{B}} T}\zeta_{i,z} \qquad \text{(Supplementary Equation 7)}$$

with potential energy in the form $U(x_i, y_i, z_i) = U_x(x) + U_y(y) + U_z(z)$ where $U_x(x)$ is similar to our 1D simulations (red curve in the inset of Supplement Fig. 8a; blue curve corresponds the tilted potential energy when maximum voltage reaches across the memristor) and $U_y(y), U_z(z)$ are both parabolic potentials modulated by short scale oscillations; green curve in the inset of Supplement Fig. 8a mimicking pinning of nanoparticles by inhomogenities/crystal-lattice[4]). Note that the overall parabolic shape of the potential is needed to prevent particles to diffuse far from the region where conducting path is formed. This also can be reached by introducing periodic boundary conditions along y and z directions, thus, keeping certain density of nano-particles. The noise is delta correlated: $\langle \zeta_{i,x}(t) \rangle = \langle \zeta_{i,y}(t) \rangle = \langle \zeta_{i,z}(t) \rangle = 0, \langle \zeta_{i,a}(0)\zeta_{j,b}(t) \rangle = \delta_{i,j}\delta_{a,b}\delta(t)$ where δ represents either Dirac or Kronecker delta and a, b takes x, y or z.

The temperature relaxation in the 3D model was still described by Newton cooling law ($\frac{dT}{dt} = \mathbb{C}_{\mathrm{T}}^{-1} Q - \kappa(T - T_0)$). However, for estimations of the resistance and the

electrical current through the memristor, we use Kirchhoff's laws assuming that the resistance between two particles *i* and *j* (green arrows in Supplement Fig. 8c is described as $R_{i,j} = R_0 e^{-\sqrt{(x_i-x_j)^2+(y_i-y_j)^2+(z_i-z_j)^2}/\lambda}$ while the resistances between *i* particle and the bottom and top memristor electrodes (shown in red and purple in Supplement Fig. 8c are estimated as $R_{i,0} = R_0 e^{-|x_i+1|/\lambda}$ and $R_{i,\mathrm{N}+1} = R_0 e^{-|x_i-1|/\lambda}$, respectively (note that ± 1 refers to the location of electrodes and all distances are normalized by half of the distance between memristor electrodes. The current Kirchhoff's law for every nano-particle can be written as $\sum_{k=0}^{i-1} I_{k,i} = \sum_{k=i+1}^{\mathrm{N}+1} I_{i,k}$ with $1 \leq i \leq \mathrm{N}$ where $I_{j,i}$ is the current from the jth nanoparticle to i^{th} nanoparticle, $I_{0,i}$ is the current from the bottom electrode and the i-nanoparticle while $I_{i,\mathrm{N}+1}$ is the current from the be i^{th} nanoparticle to the top electrode. The total current from the bottom to top electrode is defined as $I = \sum_{k=1}^{\mathrm{N}} I_{0,i}$ (note that here we ignore direct current $I_{0,N+1}$from the bottom to the top electrodes). The voltage loop Kirchhoff's law reduces to the following equations $R_{0,i}I_{0,i} + R_{i,j}I_{i,j} = R_{0,j}I_{0,j}$ for $i < j$. All these algebraic equations (for example, 21 equations for 6 particles) for currents should be solved simultaneously at each time step, thus, considerably slowing down the simulations. For this reason, we perform simulations either for six nano-particles (when building histogram, panel b) or for 9 nanoparticles (for *G*(*t*) dependence (panel a) and particle locations (panels 1-6) in the Supplementary Fig. 8.

Qualitatively, the obtained results for 3D simulations are quite similar to the results for 1D simulations: after a certain stochastic delay the resistance abruptly jumps almost to its maximum value due to the fast formation of conducting paths between electrodes (panels 1-3). The path can be bent (see panel 3), the direction of the bending is

spontaneous in our model since potentials are symmetric along y and z directions (of course in practice the bending direction can be predetermined by the device structure). When the voltage is off, the conducting path gradually dissolves and nanoparticles are re-trapped by the Ag-electrode. The distribution of the delay time also can be well fitted (Supplementary Fig. 8b by the equation obtained in Note 1). Note, that this model can be very useful for multi-terminal devices, for example, electric fields can be also applied along ether y or z directions.

Supplementary Note 4: Increase bitrate by combining diffusive memristor TRNG with a linear-feedback shift register (LFSR)

The diffusive memristor TRNG can be combined with a Fibonacci linear-feedback shift register (LFSR), which can be used to further increase the random bit generation rate. The working principal is shown in Supplementary Fig. 11. To demonstrate the feasibility, we simulated a seeded LFSR using MATLAB and diffusive memristor TRNG data. In our simulations, we used a 16-bit LFSR with feedback taps at bits 1, 2, 4, and 15[5]. To seed the LFSR, we re-seeded with 16 random bits after every 50×16 LFSR output bits. In this case, the bitrate is increased by 50 times (from 6 kbs^{-1} to 300 kbs^{-1}). We successfully produced 50 M binary bits from 1 M random bits generated by the diffusive memristor TRNG. Supplementary Table 1 shows these 50 M new generated bits also passed all 15 NIST tests without any post-processing[6]. Carefully reseeding the LFSR could be able to produce higher quality of random bits than using the TRNG alone[7]. It should also be noted that the bitrate can be further improved by engineering, such as

utilizing more-bits LFSR, although at a small cost of increased power consumption and circuit area.

Supplementary References

1. Ettelaie, E., Lishchuk, S.V. Detachment force of particles from fluid droplets, Soft Matter 11, 4251-4265 (2015).

2. Gadre, K.S., Alford, T.L. Contact angle measurements for adhesion energy evaluation of silver and copper films on parylene-n and SiO_2 substrates, J. Appl. Phys. 93, 919-923 (2003).

3. Banik, S.K., Bag, B.C., Ray, D.S. Generalized quantum Fokker-Planck, diffusion, and Smoluchowski equations with true probability distribution functions, Phys. Rev. E 65, 051106 (2002).

4. Savel'ev, S.E., Alexandrov, A.S, Bratkovsky, A.M., Williams, R.S., Molecular dynamics simulations of oxide memristors: Crystal field effects, Appl. Phys. Lett. 99, 053108 (2011).

5. Parts, R. Pseudo-Random Number Generation Routine for the MAX765x Microprocessor - Application Note – Maxim. Maximintegrated.com, 2016.

6. Rukhin, A., *et al*. A statistical test suite for random and pseudorandom number generators for cryptographic applications. NIST, Gaithersburg, MD, USA, Special Publication 800-22, 2010.

7. Koç, Ç. Cryptographic Engineering. Springer US, 2009.
